# Establishing Normative Values of Peak Expiratory Flow Rates in the Paediatric Population in Jharkhand

**DOI:** 10.7759/cureus.72439

**Published:** 2024-10-26

**Authors:** Shambhavi Datta, Sunanda Jha, Jawairia Jabeen, Ranvijay

**Affiliations:** 1 Paediatrics, Rajendra Institute of Medical Sciences, Ranchi, IND

**Keywords:** age, asthma, body mass index: bmi, children, height, jharkhand, peak expiratory flow rate (pefr), spirometry, tribal children, weight

## Abstract

Background

The peak expiratory flow rate (PEFR) is the maximum flow rate (expressed as L/s) generated during a forceful exhalation, starting from full inspiration. It is an effort-dependent parameter and reflects large airway flow. It depends on lung recoil, muscular strength of patients, and voluntary effort. The PEFR is a simple and easy way to detect early changes in lung function. The PEFR can be measured by a peak flow meter or a spirometer; however, a spirometer is more accurate than a peak flow meter. The PEFR is known to vary according to age, gender, and anthropometric parameters like height, weight, BMI, ethnicity, and altitude. PEFR values for different populations are needed because they help to determine the variable expiratory flow limitation in asthma. There is no study done to find out the normal PEFR in children in Jharkhand. Hence, this study aimed to establish the reference values of the PEFR of children in Jharkhand.

Methodology

Three hundred and sixteen healthy children belonging to the age group of 6-18 years attending the OPD of Rajendra Institute of Medical Sciences, Ranchi were studied. All the children were asked to perform spirometry, and the PEFR was recorded. Patient demographic details like age, sex, height, weight, BMI, and ethnicity were documented. These factors were selected to see how the PEFR of children in Jharkhand correlated with them and compare it with the values obtained from similar studies conducted in different parts of India.

Results

Three hundred and sixteen children were studied, consisting of 194 male participants and 122 female participants. The mean PEFR for male participants was 3.90±1.58L/s and for female participants, it was 4.04±1.49L/s. The correlation coefficient of the PEFR with height was 0.792, that of the PEFR with weight was 0.713 and that of the PEFR with BMI was 0.364. Hence, height is the best predictor of PEFR. The lowest correlation of BMI with PEFR shows that the recent rise in obesity in the paediatric population should not be expected to affect the lung function significantly.

Conclusion

The mean PEFR of children in Jharkhand is lower than that of children in northern (mean PEFR of male participants =4.41 ± 1.54 L/s, mean PEFR of female participants = 4.17 ± 1.17 L/s) and southern India (mean PEFR of male participants = 4.88±1.29 L/s, mean PEFR of female participants = 4.25±1.29 L/s). Compared to neighbouring states in eastern India, the mean PEFR of children in Jharkhand is lower.

## Introduction

A pulmonary function test is crucial for diagnosing respiratory illnesses like asthma, assessing their severity, and monitoring patient progress [1]. Various tests are available to evaluate pulmonary function, ranging from simple spirometry and peak expiratory flow to more complex and costly methods such as nitrogen wash-out, inert gas dilution, and whole-body plethysmography [2]. Each test has a specific purpose. The peak expiratory flow rate (PEFR) is particularly valuable for monitoring airway limitation as part of the Global Initiative for Asthma [1]. It is cost-effective, user-friendly, and portable. Daily PEFR evaluations can provide data to the treating physician for adjusting treatment and managing asthma effectively [3,4]. Diurnal peak flow variability of >10% in adults and>13% in children is used for diagnosis of asthma and improvement in the PEFR value is also seen as a response to therapy.

The PEFR measures the maximum flow rate during forceful exhalation, reflecting the strength of thoracoabdominal muscles. Introduced by Hadorn in 1942 and accepted as a spirometric index in 1949 [5], the PEFR is considered the simplest indicator of ventilatory function. Factors influencing the PEFR include expiratory muscle strength, airway dimensions, alveolar pressure, and lung elasticity [6]. Geographic, ethnic, socioeconomic, gender, and anthropometric factors contribute to variations in lung function among individuals.

The PEFR peaks around ages 18-20 years and remains stable until approximately 30 years in men and 40 years in women, declining with age thereafter. Test reliability can be compromised by patient cooperation issues, particularly in children, complicating interpretation. Notably, PEFR values differ between adults and children, with studies indicating lower values in the morning and higher readings in the evening.

## Materials and methods

This was conducted as a cross-sectional study within the Department of Paediatrics at the Rajendra Institute of Medical Sciences in Ranchi. Spanning from December 2022 to April 2024, the research received formal approval from the Institutional Ethics Committee, ensuring that all ethical standards were upheld throughout the process. The inclusion criteria for participation were specifically defined: only healthy children aged between 6 and 18 years whose parents provided informed consent were eligible to take part in the study.

Conversely, children were excluded if they exhibited unwillingness to participate, had experienced a recent respiratory tract infection within the last four weeks, or had a history of chronic respiratory diseases such as asthma or chronic lung disease. Additional exclusion criteria included any systemic diseases involving the lungs, underlying cardiac conditions, recent use of inhaled corticosteroids or bronchodilators, prior cardiopulmonary surgeries, or any history of tobacco smoking or substance abuse.

These criteria were applied to children who attended the Paediatrics Outpatient Department at Rajendra Institute of Medical Sciences, Ranchi and a total of 316 children were included in the study. The sampling method was convenience sampling. Detailed patient-specific data was collected, encompassing demographic information such as age, sex, anthropometric measurements, and ethnicity. To ensure accurate results, children were instructed to wear loose-fitting clothing and were given 15 minutes to relax both physically and mentally before measurements were taken. Vital parameters like heart rate, respiratory rate and blood pressure were recorded prior to the manoeuvre.

Height was measured in centimetres using a standard height stadiometer (to the nearest 1 cm), while weight was recorded in kilograms (to the nearest 0.1 kg) with a calibrated weighing scale. The PEFR was assessed with the subjects standing during outpatient department hours, which were set between 9 AM and 5 PM. The measurement, expressed in litres per second, was conducted with the aid of a spirometer. Each child received personalized instruction on the testing process, ensuring they understood the procedure. They were guided to take a deep breath and exhale forcefully into the spirometer’s mouthpiece while keeping their nostrils closed. To ensure accuracy and consistency, each child performed three PEFR measurement attempts, recognizing the need for practice and maximum effort. The highest value is recorded as the final result.

The study tools were a spirometer, a disposable mouthpiece, a stadiometer and a weighing scale.

## Results

Overall, 316 patients were included in the present study, out of which 194 were male and 122 were female. Ninety-one (28.8%) children belonged to General category, 175 (55.4%) belonged to OBC category, 28 (8.9%) belonged to ST category and 22 (7.0%) belonged to SC category. Twenty-eight were tribal children while 288 were non-tribal children. Two hundred and fifty-eight (81.6%) children practiced Hinduism, 6 (1.9%) practiced Christianity, 47 (14.9%) practiced Islam and 4 (1.3%) practiced Sarna religion. Descriptive statistics of the study group are depicted in Table 1.

**Table 1:** Descriptive statistics of study samples BMI: Body mass index; HT: Height; WT: Weight; PEFR: Peak expiratory flow rate

	Age (yr)	BMI (kg/m^2)^	HT (cm)	WT (kg)	PEFR (L/s)
Valid	316	316	316	316	316
Mean	10.39	15.517	137.93	30.354	3.9025
Std. Deviation	2.920	2.6839	16.035	10.4110	1.58950

Sex and PEFR 

The mean PEFR for male participants and female participants are depicted in Table 2. The mean PEFR of female participants in our study was significantly higher, and the p-value was <0.006.

**Table 2:** PEFR for male participants and female participants PEFR: Peak expiratory flow rate

Gender	Mean PEFR (L/s)	PEFR SD (L/s)	Frequency
Male	3.90	0.11	194 (61.3%)
Female	4.04	0.13	122 (38.6%)

Age and PEFR

**Figure 1:**
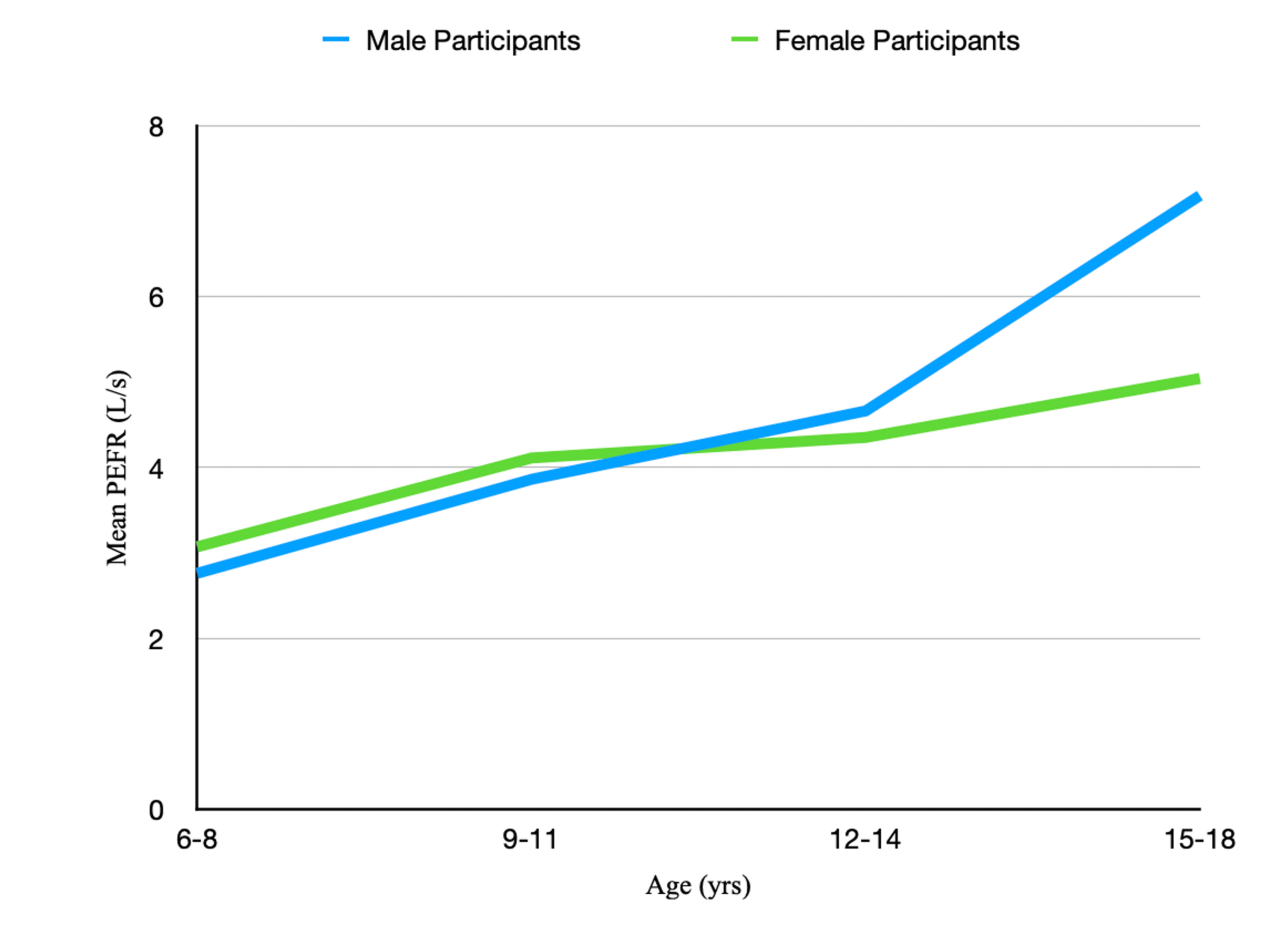
Graph depicting the mean PEFR for male participants and female participants in different age groups PEFR: Peak expiratory flow rate

The mean PEFR for different age groups are depicted in Table 3. The correlation coefficient between age and mean PEFR for the whole sample is 0.625. The correlation coefficient between age and PEFR for male participants is 0.708 and for female participants, it is 0.757. As shown in Figure 1, the mean PEFR is higher for female participants than male participants up to 11 years of age, after which the mean PEFR of male participants is higher than female participants. Beyond 12-14 years of age, the mean PEFR of female participants plateaus while the mean PEFR of male participants keeps on increasing. The difference in the mean PEFR of male participants keeps on increasing. The difference between the mean PEFR of male participants and female participants keeps on widening as age increases. A higher PEFR showed by female participants in the younger age group could be due to more physical activity in a society where a girl child is expected to assist in household duties while boys are largely gravitating toward a more sedentary lifestyle with more time spent on electronic devices.

**Table 3:** Mean PEFR across different age groups PEFR: Peak expiratory flow rate

Age group ( in years)	Frequency	Mean PEFR (L/s)	PEFR SD (L/s)
6-8	98 (31.01%)	2.81	1.31
9-11	91 (28.79%)	3.82	0.98
12-14	106 (33.54%)	4.65	1.25
15-18	21 (6.64%)	6.23	1.44

Weight and PEFR

**Figure 2:**
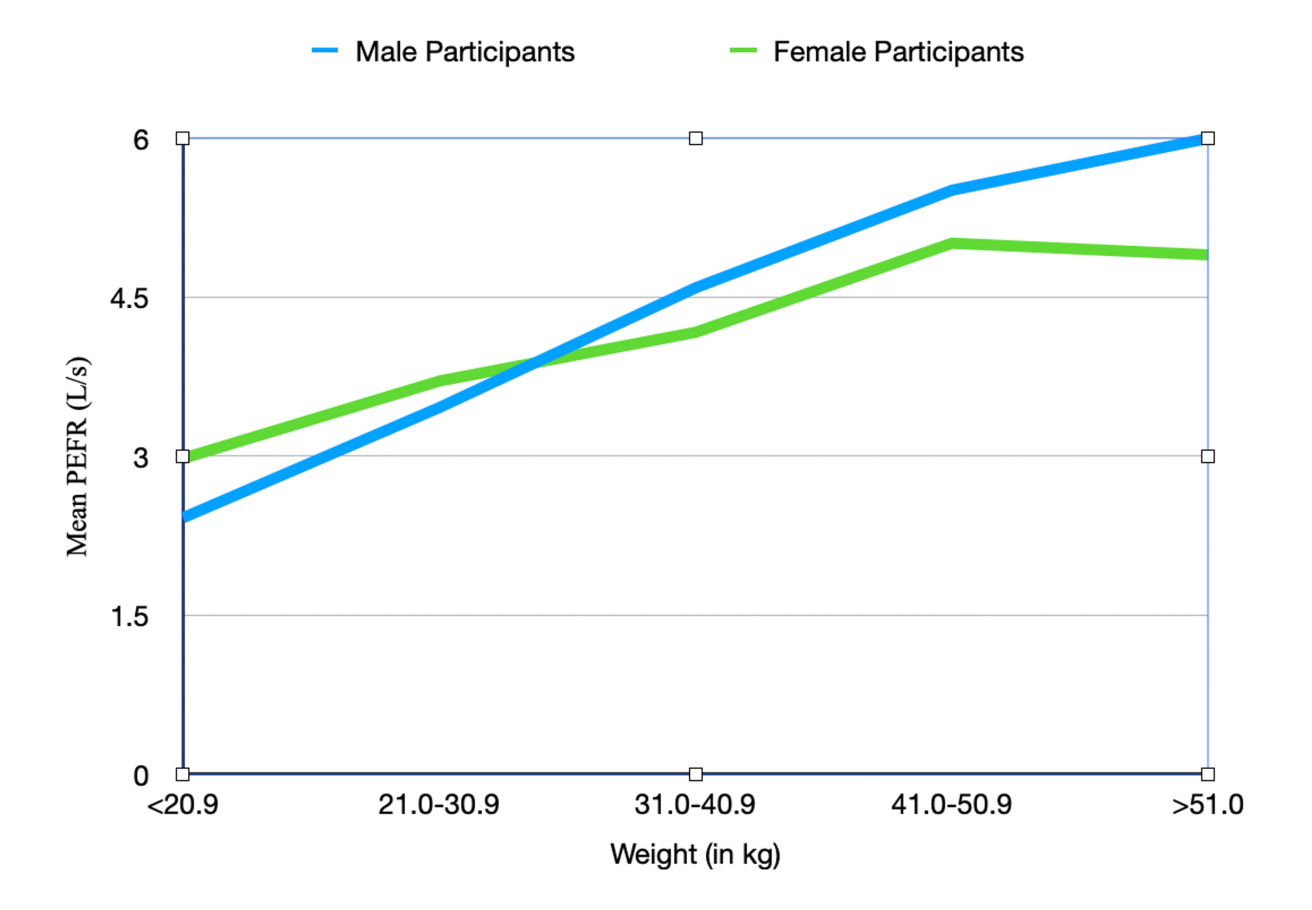
Graph depicting variation of the PEFR with weight for male participants and female participants PEFR: Peak expiratory flow rate

The mean PEFR across different weight categories is depicted in Table 4. The correlation coefficient between weight and PEFR for the whole sample is 0.713. the correlation coefficient between weight and PEFR for male participants is 0.712 and for female participants, it is 0.715. As shown in Figure 2, the PEFR increases with weight in both male participants and female participants up to 30.9 kg, after which it plateaus in female participants, while it continues to slowly increase in male participants.

**Table 4:** Mean PEFR across different weight categories PEFR: Peak expiratory flow rate

Weight group (In kg)	Frequency	Mean PEFR	PEFR SD
<20.9	56	2.44	0.69
21.0-30.9	120	3.43	1.27
31.0-40.9	85	4.55	1.10
41.0-50.9	41	5.49	1.30
>51.0	14	5.54	1.60

Height and PEFR

**Figure 3:**
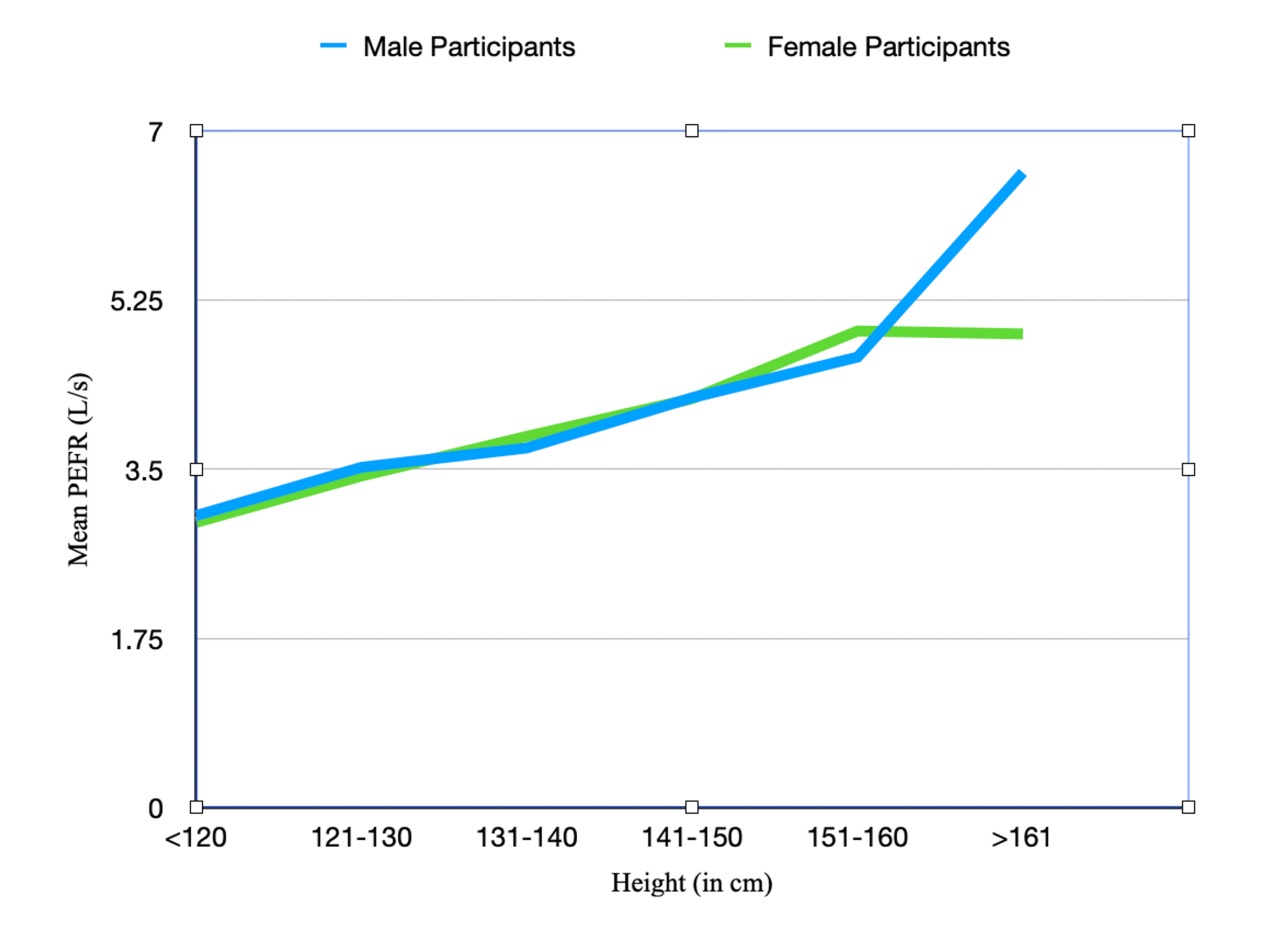
Graph showing variation in the PEFR in male participants and female participants with height PEFR: Peak expiratory flow rate

The mean PEFR across different height categories is depicted in Table 5. The correlation coefficient between height and PEFR for the whole sample is 0.792. the correlation coefficient between height and PEFR for male participants is 0.800 and for female participants, it is 0.777. As shown in Figure 3, the mean PEFR between male participants and female participants is almost equal to 150 cm beyond which the mean PEFR of male participants is higher. Beyond 150 cm, the PEFR of female participants plateaus but for male participants, it keeps on rising and the difference between male participants and female participants keeps on widening.

**Table 5:** Mean PEFR across different height categories PEFR: Peak expiratory flow rate

Height (cm) (In cm)	Frequency	Mean PEFR (L/s)	PEFR SD (L/s)
<120	55	2.38	0.63
121-130	56	2.98	0.64
131-140	58	3.79	1.54
141-150	78	4.33	1.03
151-160	48	5.29	1.09
161-170	20	6.28	1.14
171-180	1	6.75	0.12

BMI and PEFR

**Figure 4:**
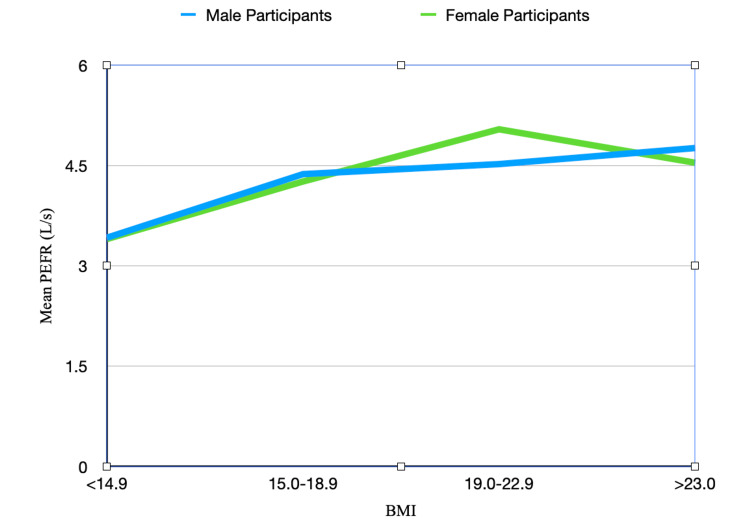
Graph depicting the mean PEFR for male participants and female participants across different BMI categories PEFR: Peak expiratory flow rate

The mean PEFR across different BMI categories is shown in Table 6. The correlation coefficient between BMI and PEFR for the whole sample is 0.364. The correlation coefficient between BMI and PEFR for male participants is 0.330 and for female participants, it is 0.398. As shown in Figure 4, mean PEFR continues to increase with BMI in both male participants and female participants till around 18.9 kg/m^2^ after which it decreases with further increase in BMI in both male participants and female participants.

**Table 6:** Mean PEFR across different BMI categories PEFR: Peak expiratory flow rate

BMI (kg/m^2^)	Frequency	Mean PEFR (L/s)	PEFR SD (L/s)
<14.9	162	3.48	1.46
15.0-18.9	121	4.33	1.44
19.0-22.9	25	4.84	1.64
23 & above	8	4.77	1.44

Ethnicity and PEFR

The mean PEFR for tribal children and non-tribal children is shown in Table 7. The difference in the PEFR of tribal children and non-tribal children is statistically significant (p-value 0.000).

**Table 7:** Mean PEFR for tribal children and non-tribal children PEFR: Peak expiratory flow rate

Ethnicity	Mean PEFR (L/s)	PEFR SD (L/s)
Non-tribal	3.89	1.51
Tribal	4.60	1.83

## Discussion

The study aimed to establish the normal PEFR values for children in Jharkhand, which could serve as a reference for healthcare providers. Assessing lung function is crucial in respiratory medicine, both for healthy individuals and those with respiratory conditions. PEFR, an effort-dependent parameter, originates from the large airways within approximately 100-120 milliseconds of forced expiration onset, reaching its peak within 10 milliseconds. Recently, the PEFR has gained significance as a widely used method for evaluating obstructive and restrictive lung diseases. Given the numerous sources of variability in pulmonary function, regional norms are essential. Factors such as airway resistance, voluntary effort, and potential compression of thoracic airways contribute to intra-individual variability. Geographical factors, environmental and occupational exposures, and socioeconomic status also influence within-individual variation [7]. Inter-individual variation is influenced by factors like height, weight, age, race, and health history. Having local reference values is especially important for the state of Jharkhand as it has abundant mining activity going on which makes children more prone to developing respiratory problems due to exposure to mining dust. Local standard data aids in early detection of changes and response assessment in asthmatic children.

Our study found a significant positive correlation (p < 0.0001) between PEFR and age, weight, height, BMI, and ethnicity, with height showing the highest correlation (r = 0.791). A study by Deo et al. (2016) in Sasaram district, Bihar, a neighbouring state, reported lower mean PEFR values compared to our findings [8]. Similarly, Chidambaram et al. (2016) in Bhilai, Chattisgarh, found higher mean PEFR values than those observed in Jharkhand [9]. Garg et al. (2015) studied PEFR in Ghaziabad, Uttar Pradesh, reporting higher mean values than those in Jharkhand across all height categories [7]. Choudhuri et al. (2015) conducted a study in Tripura, noting comparable PEFR values between tribal and non-ethnic Bengali children, but higher mean values than those in Jharkhand [10]. Chatterjee and Mandal (1991) in West Bengal found higher PEFR values compared to Jharkhand [11]. Manjreeka et al. (2014) evaluated tribal children in Odisha, reporting lower mean PEFR values than those of tribal children in Jharkhand [12]. Sangwan et al. attempted to determine normal PEFR in children of Solan, Himachal Pradesh and showed PEFR values for children residing in Solan to be higher than that found in our study [13]. This may be due to the elevation of Solan which is 1550 metres (5090 ft) above sea level while Ranchi is 651 metres (2140 feet) above sea level. Values of PEFR of local Jharkhand children were also found to be lower than those of rural school-going children residing in Bellur, Karnataka as shown by a study done by Manjunath et al. in 2013 [14]. Mittal et al. (2010) determined the mean PEFR of children in Patiala, which is at an elevation of 257 metres (820 ft) above sea level. Values obtained for male participants in Patiala were higher than those in our study while the mean PEFR of female participants in Patiala was lower than the values obtained in our study. This difference could be due to the mean height of male participants in our study (136 cm) being lower than that of female participants (139cm) [15]. Bhanbro et al. (2022) conducted a study to evaluate PEFR in Khairpur Mir’s city of Pakistan among children aged 6-12 years. Their study found that the mean PEFR of boys was higher than that of girls of the same age but it was not statistically significant while the mean PEFR of girls in our study was higher than that of boys and it was statistically significant [16]. 

Our study has several weaknesses which include an asymmetrical age distribution with the majority belonging to the 12-14 years age group. The PEFR was recorded both in the morning and afternoon. Several studies have found that the lowest PEFR values are seen in the morning and the highest in the evening. Subjects were unevenly distributed across height and weight categories. To address this, increasing the sample size and randomly selecting subjects from the general population, rather than exclusively from healthy children attending the Department of Paediatrics OPD, would have been beneficial. Some subjects experienced challenges in performing the manoeuvre, resulting in lower values despite numerous demonstrations and repeated practice attempts by the subjects.

## Conclusions

The PEFR is of immense importance in paediatric respiratory medicine practice as it is a tool to identify obstructive airway diseases. It has been used as a criterion to determine variable airflow limitation in asthma. Establishing region-specific reference values for the PEFR is crucial. Having local reference values instead of relying on standard values derived from studies done in children with different demographics will inevitably lead to misdiagnosis. Our study demonstrated that normal PEFR values in children vary significantly based on age, sex, weight, height, BMI, ethnicity, altitude, and regional factors. Our study showed a significantly higher PEFR for tribal children compared to non-tribal children. We found a positive correlation between PEFR and anthropometric factors such as height, weight, and BMI. 

Our statistical analysis identified significant correlations between the PEFR and variables such as height, age, BMI, and weight across all groups. Age data, possibly influenced by parental input in school records, may introduce bias. Consistent with previous research, our study confirms a strong correlation between the PEFR and height, supporting the use of height-based predictive equations. Additionally, boys consistently exhibited higher PEFR values compared to girls at equivalent heights, consistent with prior findings. The mean PEFR for female participants is higher than that of male participants up to 30.9 kg, beyond which the mean PEFR of male participants is higher than that for female participants. The difference between the PEFR of male participants and female participants keeps on increasing beyond this point (i.e. 30.9kg). The mean PEFR between male participants and female participants is almost equal to 150 cm beyond which the mean PEFR of male participants is higher. Beyond 150 cm, the PEFR of female participants plateaus but for male participants, it keeps on rising and the difference between male participants and female participants keeps on widening. The mean PEFR of male participants and female participants is almost equal to BMI of 18.9 kg/m^2^. The PEFR for female participants peaks at around 19.0-22.9 kg/m^2^ following which it decreases beyond 23 kg/m^2^. We hope our data sheds some light on how the geographical factors of Jharkhand affect the PEFR in children and help clinicians in this part of our country. This study can also pave the way for future studies where the role of socioeconomic status and food habits in influencing the PEFR could be explored. Another prospect is to see how the PEFR varies across various tribal groups in Jharkhand.

## Appendices

**Table 8:** Case record form

Case No.	
Demographic Details of the Participant	
Name of the Participant	
Father’s Name	
Age	
Sex	
Ethnicity (Tribal/ Non-tribal)	
Religion	
Category (General/OBC/ST/SC)	
Address	
Mobile no.	
Clinical Details of the Participants	
OPD ID	
Date of Registration	
Chief Complaints with Duration	
Past Illness	
Treatment History	
Contact History	
Birth History	
Immunisation History	
Developmental History	
Personal History	
Family History	
Socioeconomic History	
General Examination	
Examination of Respiratory system	
Examination of Cardiovascular system	
Examination of Gastrointestinal system	
Examination of Central Nervous system	
Anthropometry	
Height (cm)	
Weight (kg)	
BMI (kg/m^2^)	
PEFR (L/s)	
